# HomoplasyFinder: a simple tool to identify homoplasies on a phylogeny

**DOI:** 10.1099/mgen.0.000245

**Published:** 2019-01-21

**Authors:** Joseph Crispell, Daniel Balaz, Stephen Vincent Gordon

**Affiliations:** ^1^​ School of Veterinary Medicine, College of Health and Agricultural Sciences, University College Dublin, Republic of Ireland; ^2^​ Royal (Dick) School of Veterinary Studies, University of Edinburgh, Edinburgh, Scotland

**Keywords:** HomoplasyFinder, homoplasy, phylogenetic, convergence, Java, R package

## Abstract

A homoplasy is a nucleotide identity resulting from a process other than inheritance from a common ancestor. Importantly, by distorting the ancestral relationships between nucleotide sequences, homoplasies can change the structure of the phylogeny. Homoplasies can emerge naturally, especially under high selection pressures and/or high mutation rates, or be created during the generation and processing of sequencing data. Identification of homoplasies is critical, both to understand their influence on the analyses of phylogenetic data and to allow an investigation into how they arose. Here we present *HomoplasyFinder*, a java application that can be used as a stand-a-lone tool or within the statistical programming environment R. Within R and Java, *HomoplasyFinder* is shown to be able to automatically, and quickly, identify any homoplasies present in simulated and real phylogenetic data. *HomoplasyFinder* can easily be incorporated into existing analysis pipelines, either within or outside of R, allowing the user to quickly identify homoplasies to inform downstream analyses and interpretation.

## Data Summary

Three previously published datasets were used in the current analyses:

1. Crispell *et al.* 2017: Whole Genome Sequence *Mycobacterium bovis* data published under bioproject accession number: PRJNA363037 on National Centre for Biotechnology Information (NCBI).
2. Grandjean *et al.* 2017: Whole Genome Sequence *Mycobacterium tuberculosis* data published under project: ERP004677 on European Nucleotide Archive (ENA).
3. Didelot and Wilson 2015: Whole Genome Sequence *Staphylococcus aureus* published as an example dataset for *ClonalFrameML*: http://www.danielwilson.me.uk/files/ClonalFrameML/

All the code generated for this manuscript and for *HomoplasyFinder* is freely available on GitHub. General scripts are here, the Java source code files are available here, and the R package (*homoplasyFinder*) can be accessed here.

**Impact Statement**

We are currently in a sequencing revolution; sequencing data is being generated and analysed at an unprecedented rate. Often phylogenetic approaches, which study the ancestral relationships between sequences, underpin the analysis of sequencing data. A homoplasy is a character shared across clades in a phylogeny that don’t share direct ancestry, are an indication of inconsistency between the phylogenetic tree and the sequences used to build it. Homoplasies on a phylogeny can be created naturally, from convergent evolution or recombination, or unnaturally, during the generation and processing of sequencing data. Before the phylogenetic relationships in a tree are interpreted, it is crucial that any inconsistencies between the sequencing data and the tree are identified. Here we present *HomoplasyFinder* an open-source tool that will automatically identify all homoplasies present on a phylogeny. Implemented in Java and accessible in the command line, in R, or via a graphical user interface, *HomoplasyFinder* is a fast and easy-to-use tool that can be readily incorporated into existing phylogenetic analyses pipelines.

## Introduction

A phylogenetic tree describes the ancestral relationships between nucleotide sequences. The accuracy of the ancestral relationships depicted in the phylogenetic tree relies upon the vast majority of nucleotide differences, which define the tree, resulting from substitution events that only occurred once [1]. A homoplasy defines when the same substitution occurs multiple times independently in separate evolutionary lineages [2–4].

Homoplasies often obscure the true evolutionary history of sequences by suggesting greater genetic similarity. The presence of a large number of homoplasies in a set of sequences can therefore obscure their true phylogenetic relationships [5, 6].

There are multiple ways that homoplasies can arise. Convergent evolution can result in the same nucleotide evolving independently at a position on a genome [7]. Widespread antibiotic use is known to increase selective pressures, prompting convergent evolution that can promote resistance [8, 9]. Recombination, defined as the reorganising of the genome or the incorporation of novel genetic material into it [10, 11], can also create homoplasies [12]. In addition, homoplasies can be introduced during the generation or processing of sequencing data [13, 14].

In 1971, Fitch [15] described calculating the minimum number of state changes required on a phylogenetic tree to explain the characters observed for that state at the tips of the tree. An example of a state is a nucleotide at a position in an alignment, that state can vary in different sequences and is usually one of four nucleotides (A=Adenine, C=Cytosine, G=Guanine, and T=Thymine). The consistency index uses the minimum number of state changes to determine how consistent the nucleotides observed at a site in an alignment are with a phylogeny [16]. When the consistency index is equal to one that site is entirely consistent, values less than one indicate a degree of inconsistency, with zero meaning completely inconsistent.

The consistency index can be used to efficiently identify homoplasies. It is possible to calculate the consistency index using a variety of tools, for example: *phangorn* [17], *phylip* [18], *treetime* [19] and *mesquite* [20], and in *RAxML* [21]. How to access these tools varies considerably, requiring differing expertise: *phangorn* is a package within the statistical programming environment R, *phylip*, *treetime* and *RAxML* work in the command line, and *mesquite* provides a Graphical User Interface (GUI). These tools are designed to conduct varied and complicated phylogenetic analyses, but not specifically for identifying homoplasies.

Here, we present *HomoplasyFinder* a tool specifically designed to identify homoplasies. Using the consistency index, *HomoplasyFinder* is able to quickly identify homoplasies given a phylogenetic tree and nucleotide alignment. Once identified, *HomoplasyFinder* returns an annotated Newick formatted phylogeny, a simple report and a nucleotide alignment without any inconsistent sites. *HomoplasyFinder* is an open-source Java application that can be accessed in R, in the command line or via a GUI. The current article describes the implementation and testing of *HomoplasyFinder*.

## Theory and implementation

### Calculating consistency index

**Fig. 1.**
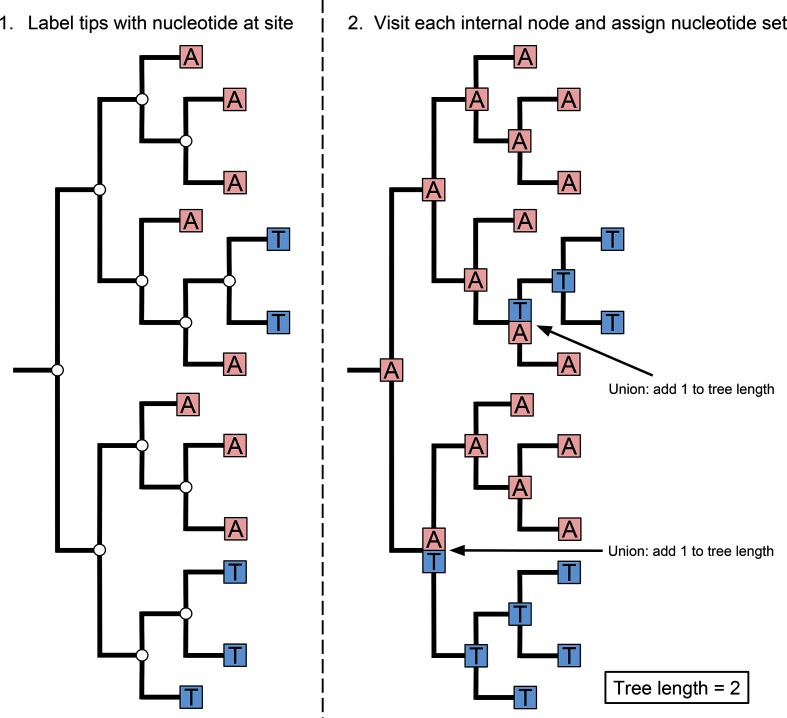
Diagrams demonstrating calculating the tree length of one site in a nucleotide alignment. Step 1. Demonstrates how the nucleotides at one site in each sequence are assigned to the tips in a phylogeny. Step 2. Demonstrates defining the nucleotide sets at each internal node, as either the union or intersection of the nucleotide sets of the descendent nodes, and calculating the tree length.

The algorithm used to calculate the minimum number of changes for the characters at each alignment site on a phylogenetic tree was adapted from that of Swofford *et al.* [22] (summarised in Fig. 1). The algorithm uses the following steps:

1. Read Newick file (A Newick file is a standard file format for storing phylogenetic trees)
2. Read FASTA file (A FASTA file is a standard file format for storing a set of unaligned or aligned nucleotide sequences)
3. Initialise a vector of zeros with length equal to the number of sites in the alignment to record the tree length for each site
4. Assign the nucleotide sequences to their respective tips in the phylogenetic tree. Note that these sequences are represented as character sets for each site in the alignment.
5. Select an unvisited internal node. If none is available, finish.
  - Check if any descendant nodes are unvisited. If they are, visit them first.
  - Examine each site in the alignment.
    - If the character sets for each descendant node at the current site have elements in common - assign the *intersection* of the character sets to the current internal node for the current site. Otherwise, assign the *union* of the character sets and increment the tree length for the current site
  - Set the current internal node to visited and return to step 5

The consistency index for each site in the alignment is then calculated by dividing the minimum number of changes on the phylogeny by the number of different nucleotides observed at that site minus one. Sites with a consistency index of less than one will be reported as inconsistent and potentially homoplasious.

Importantly, where *N*s are present in the alignment (an *N* indicates that insufficient data are available to determine the nucleotide at that sequence position), the character set will contain four nucleotides (A, C, G and T). Similarly, other IUPAC (International Union of Pure and Applied Chemistry) codes can be handled.

The steps described were implemented in Java, available here. All the source code is hosted on the open-source platform github. *HomoplasyFinder* is licensed under a GPL-3.0 licence. The R package, *homoplasyFinder*, uses the R package *rJava* [23] to interact with the *HomoplasyFinder* Java application. Importantly, *HomoplasyFinder*, and the consistency index in general, will be most accurate when the phylogeny is well-resolved. In addition, *HomoplasyFinder* is designed to work on a Newick formatted phylogeny, which will be rooted.

### Testing HomoplasyFinder

#### Identifying homoplasies in simulated data

*HomoplasyFinder* was tested using simulated nucleotide sequence alignments with known homoplasies created in R (v3.4.0 [24]). A continuously mutating sequence of nucleotides was sent through a simulated population, passing from infected to susceptible individuals. Mutations were modelled using a Poisson distribution, occurring, on average, once every two time-steps and at unique positions. The infectiousness of each infected individual per time-step was 0.001 (on average, one in every 1000 susceptible individuals were infected by a single infected individual). Each infected individual carried their own continuously mutating sequence of nucleotides, which they received from the individual that infected them. Infected individuals were sampled (removed and their sequence recorded) throughout each simulation with a probability of 0.05 (per infected individual per time-step). Each simulation used 200 individuals and ran until 100 individuals were sampled. A maximum likelihood phylogenetic tree was reconstructed with the sampled sequences from each simulation using the *phangorn* R package [17].

Homoplasies were introduced into the simulated sequences by randomly selecting a pair of non-nested nodes (*i* and *j*) on the maximum likelihood phylogenetic tree. A random position with a nucleotide that was unique to the sequences of node *i* was selected without replacement and assigned to node *j’*s sequences. The simulated nucleotide sequences with their inserted homoplasies were stored in a FASTA file, and a maximum likelihood phylogenetic tree was reconstructed and stored in a Newick file.

An additional set of simulations were completed to investigate how recombination events influence the identification of homoplasies using *HomoplasyFinder.* A varying number of recombination events were incorporated into the simulated sequences with 100 previously inserted homoplasies, and the number of the inserted homoplasies identified by *HomoplasyFinder* was recorded. Similarly to the insertion of homoplasies, recombination events were simulated by randomly selecting a pair of non-nested nodes (*i* and *j*) on the phylogenetic tree reconstructed using simulated sequences. Next, a 100 bp region (*r*) of the simulated alignment was randomly chosen. Lastly, the consensus sequence for the region *r* from node *i*’s sequences was assigned to node *j*’s sequences.

#### Identifying homoplasies in published data

Three whole-genome-sequence datasets, *Mycobacterium bovis* data (298 genomes) published by Crispell *et al.* [25], *M. tuberculosis* data (472 genomes) published by Grandjean *et al.* [26], and *Staphylococcus aureus* data (110 genomes) published by Didelot and Wilson [27], were processed according to the methods described in the respective articles. *HomoplasyFinder* was used to check whether any homoplasies were present in these data. Grandjean *et al.* [26] published a list of a subset of the homoplasies they identified and these were compared with the output from *HomoplasyFinder. ClonalFrameML* [27] was used to analyse the *S. aureus* data and its output was compared with the results from *HomoplasyFinder*.

#### Comparing to published tools

*Treetime* and *phangorn* were selected for speed comparisons with *HomoplasyFinder*. Whilst *phangorn* directly calculates the consistency index, *Treetime* uses ancestral sequence reconstruction methods to identify homoplasies. The time taken for *HomoplasyFinder* (accessed within R and in the command line) to recognise simulated homoplasies was compared to the same data analysed by *Treetime* and *phangorn*. The comparisons were carried out on a desktop Ubuntu computer with an AMD Ryzen 5 1600X processor with six cores and 16 GB of Random Access Memory (RAM).

### Using HomoplasyFinder

*HomoplasyFinder* can be accessed within R, in the command line or via a GUI. The R package, *homoplasyFinder*, can be directly imported into a package library using the following commands:

install.packages(‘devtools’)

library(‘devtools’)

install_github(‘JosephCrispell/homoplasyFinder’)

library(‘homoplasyFinder’)

Once the package is loaded, the runHomoplasyFinderInJava() function can be used to execute *HomoplasyFinder*.

In the command line the application without a GUI can be ran using:

java -jar homoplasyFinder.jar - -fasta fastaFile - -tree treeFile where the full paths are provided to FASTA and NEWICK tree files. A range of command line options are available and can be viewed using the - -helpflag. The application with a GUI can be executed by double clicking.

*HomoplasyFinder* can produce three different output files: a report (in a CSV format) detailing the consistency information calculated for each homoplasious site in the input alignment (use the - -includeConsistent flag to include information for all sites), an alignment without the inconsistent sites (in a FASTA format), and an annotated phylogeny (in a NEWICK format). These output files are all in standard formats and as such should be accessible to any programs designed to handle these formats, for example the annotated phylogeny can be viewed within R using the *ape* package, or by using *figtree* or *icytree*. Full documentation for *HomoplasyFinder* can be found here.

## Results and discussion

### Identifying simulated homoplasies

**Fig. 2.**
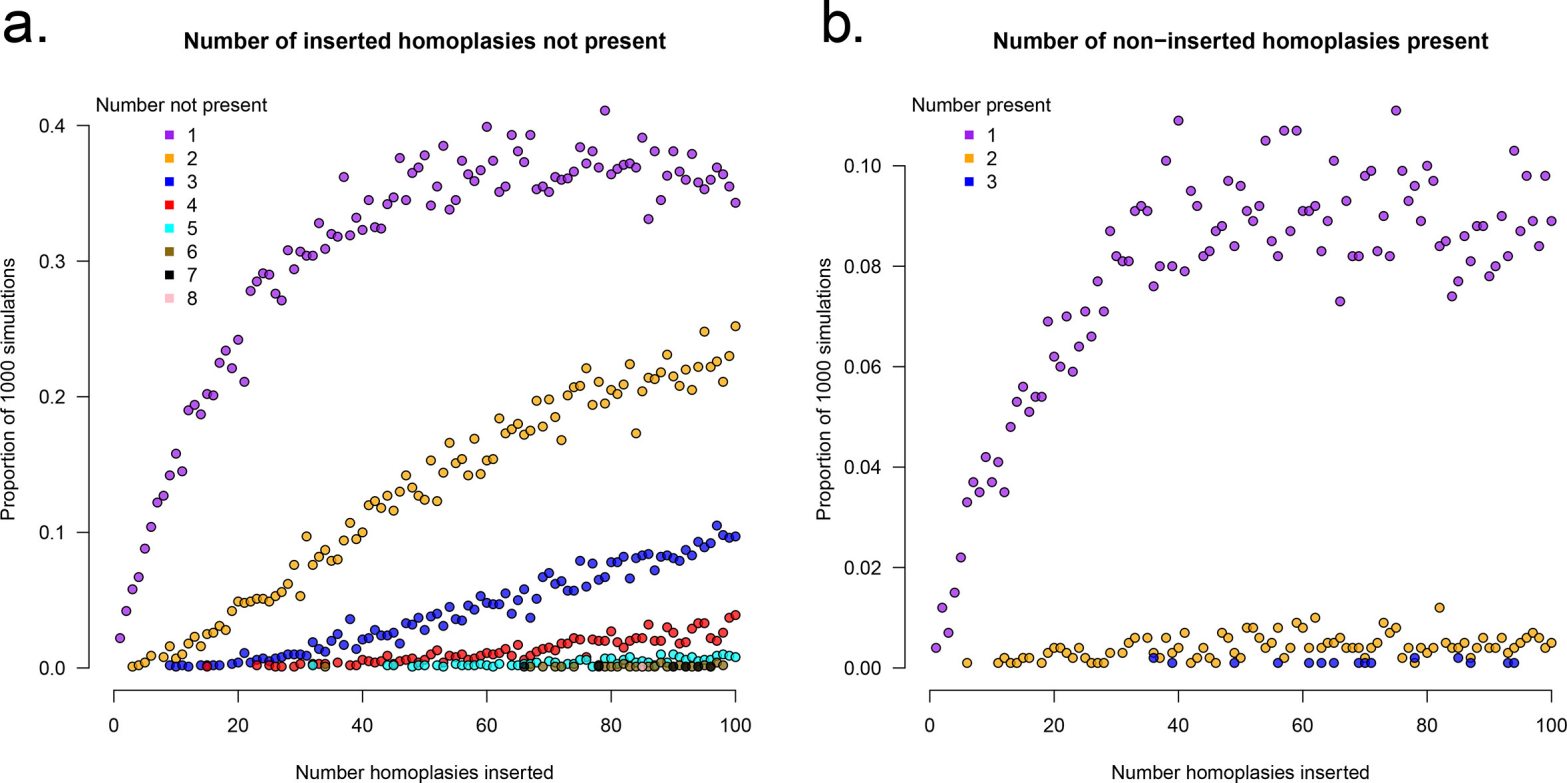
Identifying homoplasies in simulated data using *HomoplasyFinder*. (a) The proportion of 1000 simulated phylogenetic datasets with X inserted homoplasies (where the number of inserted homoplasies ranged from 0 to 100 in steps of 1) not identified using *HomoplasyFinder* - i.e., false negatives. (b) The proportion of 1000 simulated phylogenetic datasets with X non-inserted homoplasies identified by *HomoplasyFinder* - i.e., false positives. Each point is coloured according to X, which represents either the number of inserted homoplasies not found, or the number of non-inserted homoplasies found.

**Fig. 3.**
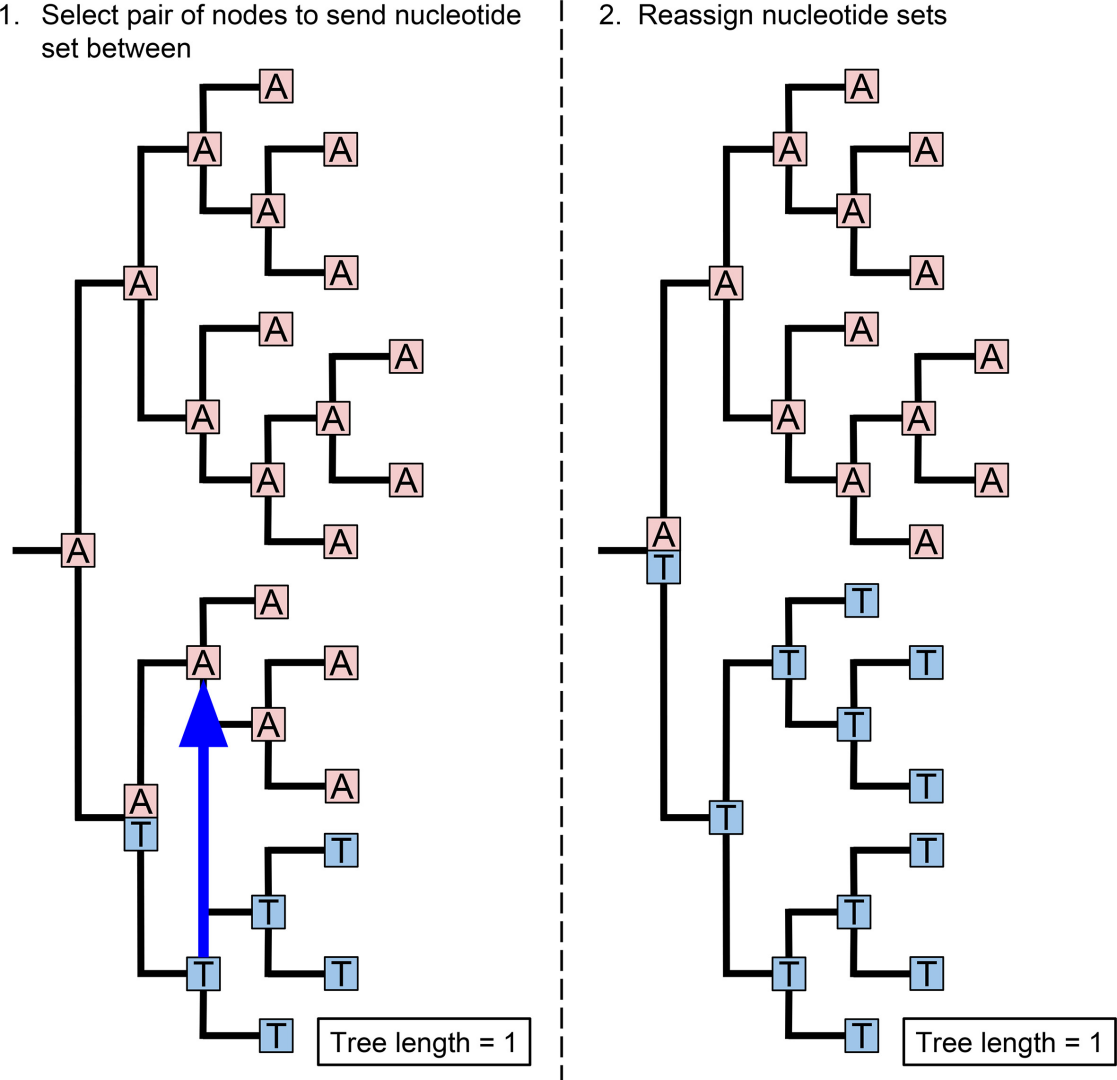
Creating non-identifiable homoplasies. An example of how the process of creating simulated, and naturally evolving, homoplasies can result in homoplasies that can’t be detected.

**Fig. 4.**
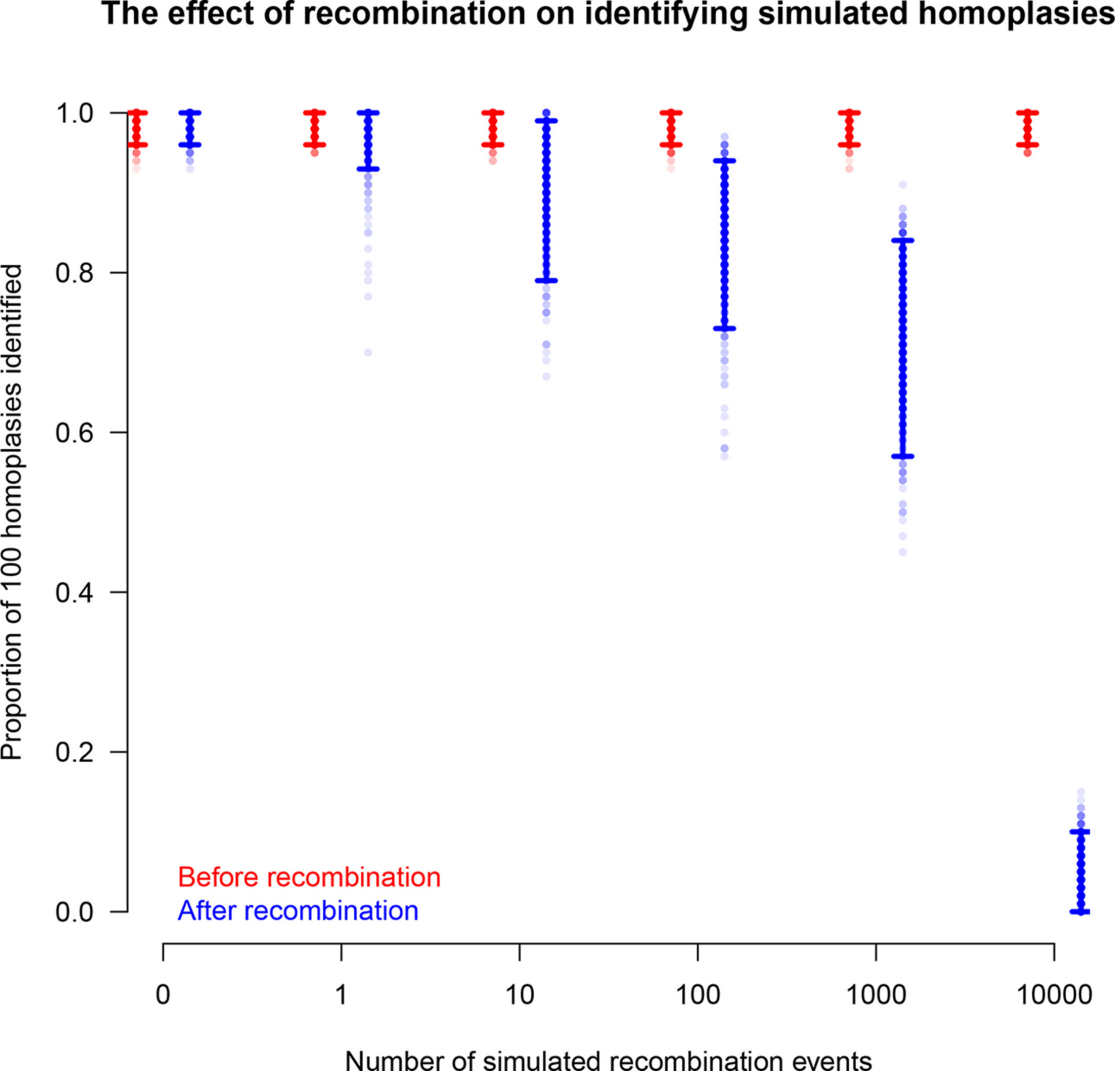
The proportion of 100 homoplasies inserted into simulated nucleotide sequences that were identified by *HomoplasyFinder* before (red) and after (blue) recombination events had been applied to the sequences. The simulated sequences had either 0, 1, 10, 100, 1000 or 10 000 recombination events applied to them. The vertical lines represent the range between the lower 2.5 % and upper 97.5 % of the proportions calculated on each of 1000 replicates.

*HomoplasyFinder* was able to accurately identify the homoplasies within the simulated phylogenetic datasets (Fig. 2a). On average across the simulations, *HomoplasyFinder* detected 98.5 % (Lower 2.5 %: 92.4 %, Upper 97.5 %: 100 %) of the inserted homoplasies. Simulated homoplasies that weren’t detected were created when the sequences involved were (or ended up being) directly adjacent on the phylogenetic tree (Fig. 3). In addition, Fig. 2b demonstrates that the process of inserting homoplasies can alter the phylogenetic data such that non-inserted homoplasies were present. Fig. 4 demonstrates that recombination events can strongly influence the structure of the phylogeny and thereby the ability to accurately detect homoplasies. Recombination events themselves can cause homoplasies. Therefore, if high levels of recombination are present in genetic data, their influence on the phylogeny must be accounted for prior to identifying homoplasies.

Whilst the simulations demonstrate that *HomoplasyFinder* can accurately identify homoplasies in phylogenetic data, they also highlight the circularity to the identification of homoplasies. The sequence data with homoplasies is used to reconstruct the phylogenetic tree that is used to identify the homoplasies. A phylogenetic tree is necessary to identify homoplasies as it defines hierarchical clusters of the sequences. Therefore, the accuracy of homoplasy identification relies upon the homoplasies themselves having not strongly influenced the true phylogenetic relationships between the sequences.

### Identifying homoplasies in published data

*M. bovis* and *M. tuberculosis* are both slowly evolving, highly conserved pathogens with relatively large genomes that rarely recombine [28, 29]. Therefore, genome sequence data of these pathogens should provide a phylogenetic signature that is close to perfect (without homoplasies), provided the sequencing quality is good.

**Fig. 5.**
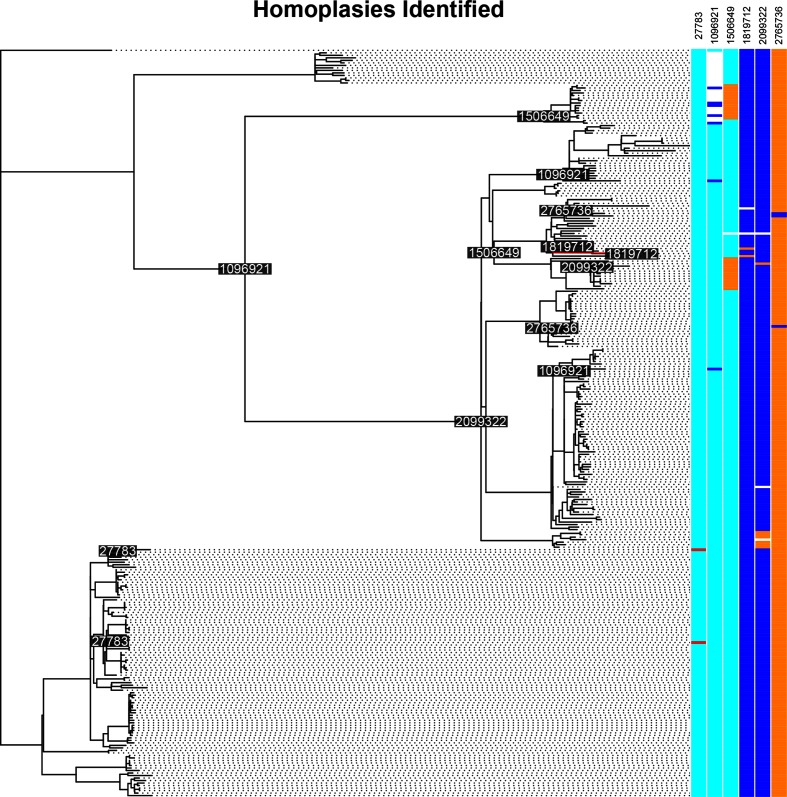
Identifying homoplasies in *M. bovis* data using *HomoplasyFinder*. A phylogenetic tree reconstructed using 298 published *M. bovis* whole genome sequences [25]. *HomoplasyFinder* identified homoplasies at six different positions (0.2 % of the 3852 polymorphic positions identified), the nucleotides associated with these positions in each sequence are plotted and coloured according to their type (Adenine=red, Cytosine=blue, Guanine=cyan and Thymine=orange). Where no information for the nucleotide at a particular site in a sequence was available, it is coloured white. The positions, on the *M. bovis* reference genome [33], associated with the identified homoplasies are reported in the top right and annotated on the internal nodes where a change was necessary. To avoid overlapping one of the labels was slightly moved and a red line points to the node it annotates.

*HomoplasyFinder* identified six homoplasies (0.2 % of the 3852 polymorphic sites identified) present in the alignment analysed and published by Crispell *et al.* [25] (Fig. 5). Given the stringent quality filtering carried out by Crispell *et al.* [25], and that each of the homoplasies identified was found in three or more sequences, these are likely to be the result of evolutionary, rather than sequencing, processes.

Three of the six homoplasies identified in the *M. bovis* data are found close to, or within, genes encoding cell-surface proteins that are known to be highly variable [30, 31]. In addition, the homoplasy found at position 1 505 649 was associated with two large independent clades and, therefore, is likely to have resulted from convergent evolution as a result of the high selection pressures exerted on genes encoding surface proteins. The presence of these six homoplasies is unlikely to have strongly influenced the structure of the published phylogeny, but these sites, and their influence, should be considered in any future research.

*HomoplasyFinder* identified 798 homoplasies (3.6 % of the 21 877 polymorphic sites identified) in the *M. tuberculosis* data published by Grandjean *et al.* [26]. The high number of homoplasies identified is likely to have resulted from the quality filtering carried out by Grandjean *et al.* [26] being less stringent than that of Crispell *et al.* [25]: no proximity filtering was conducted, difficult to sequence regions were retained (such as repeat regions and mobile elements), and the quality thresholds were lower (for example, no read depth filter was used and a high-quality base depth was set to two). Grandjean *et al.* [26] called variants and compared them with phenotypic drug resistance profiles, making specificity of the variants called less important, therefore the low quality thresholds used here are unlikely to have caused problems in their downstream analyses. Of the 105 homoplasies reported by Grandjean *et al.* [26], 11 weren’t identified by *HomoplasyFinder*, or *phangorn*. These 11 sites not identified by *HomoplasyFinder* were associated with poor quality sequence data and showed no evidence of being homoplasious upon manual examination.

*ClonalFrameML* identified 1518 recombination events in the *S. aureus* dataset analysed here and reported 13 743 homoplasious patterns (to save computation, *ClonalFrameML* condenses the input alignment into unique patterns (*n*=20 791) that can be linked back to individual positions). These homoplasious patterns were all identified by *HomoplasyFinder* and found to be associated with 19 810 positions in the nucleotide alignment. Of these 19 810 positions, 12 097 were found within the regions of the alignment that were associated with recombination events. For *S. aureus*, which undergoes high levels of recombination [32], in order to reconstruct a well-resolved phylogeny the effects of recombination must be considered. Methods to account for recombination include reconstructing the phylogeny using areas of the genome that are free from recombination or using tools such as *ClonalFrameML* that can account for recombination whilst reconstructing a phylogeny.

### Comparison with published tools

**Fig. 6.**
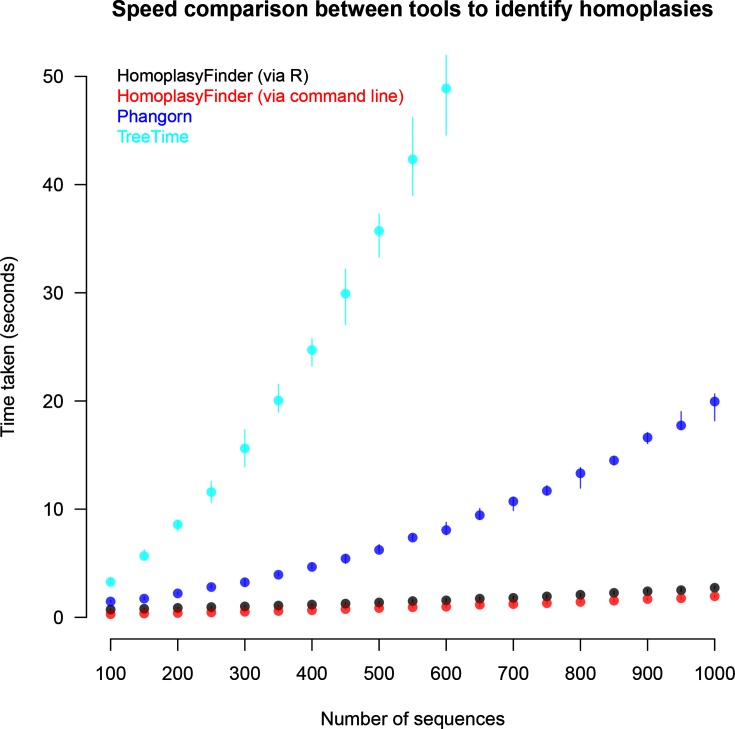
Comparing *HomoplasyFinder* to *phangorn* and *treetime*. The time taken to identify 10 homoplasies present in simulated phylogenetic datasets by *HomoplasyFinder* accessed in R and the command line, *phangorn* in R and by *treetime* in the command line. A total of 190 different datasets were tested, ranging from 100 to 1000 sequences, in steps of 50 with 10 replicates of each. The number of positions in these sequences ranged from 4000 to 20 000. The points and vertical lines plotted represent the mean, and range, respectively, of the ten replicates.

*HomopasyFinder*, accessed via Java directly or using R, was faster than both *phangorn* and *treetime* (Fig. 6). *HomoplasyFinder* analysed the largest dataset of 1000 sequences each containing approximately 20 000 nucleotides, in less than five seconds, whilst *phangorn* took around 20 s and *treetime* took over two minutes. During these trials as well as identifying homoplasies, *HomoplasyFinder*, in contrast to the other tools, was also creating a Newick file containing an annotated phylogeny and a new FASTA file with an alignment without the sites where homoplasies were identified.

### Conclusions

Here, we describe *HomoplasyFinder*; an open-source, non-parametric Java application that is accessible within R, in the command line, and via a GUI. *HomoplasyFinder* uses the consistency index to quickly and accurately identify homoplasies. Once *HomoplasyFinder* has been used, any homoplasies identified can be interrogated, and their origins and influence on future work determined.

## Data bibliography

1. HomoplasyFinder software and source code: https://github.com/JosephCrispell/homoplasyFinder.
